# Transcriptomic response of wolf spider, *Pardosa pseudoannulata*, to transgenic rice expressing *Bacillus thuringiensis* Cry1Ab protein

**DOI:** 10.1186/s12896-016-0325-2

**Published:** 2017-01-18

**Authors:** Juan Wang, Yuande Peng, Kaifu Xiao, Baoyang Wei, Jilin Hu, Zhi Wang, Qisheng Song, Xuguo Zhou

**Affiliations:** 1 College of Bioscience and Bitechnology, Hunan Agriculture University, No1 Nongda Road, Changsha, 410128 Hunan China; 2 Institute of Bast Fiber Crops, Chinese Academy of Agricultural Sciences, Changsha, Hunan 410205 China; 3 Division of Plant Sciences, University of Missouri, Columbia, MO 65211 USA; 4 Department of Entomology, University of Kentucky, Lexington, KY USA

**Keywords:** *Pardosa pseudoannulata*, Cry1Ab, Development, RNA-Seq, Chitin, Cuticle

## Abstract

**Background:**

*Bacillum thuringiensis* (*Bt*) toxin produced in *Cry1*-expressing genetically modified rice (Bt rice) is highly effective to control lepidopteran pests, which reduces the needs for synthetic insecticides. Non-target organisms can be exposed to *Bt* toxins through direct feeding or trophic interactions in the field. The wolf spider *Pardosa pseudoannulata,* one of the dominant predators in South China, plays a crucial role in the rice agroecosystem. In this study, we investigated transcriptome responses of the 5th instar spiders fed on preys maintained on Bt- and non-Bt rice.

**Results:**

Comparative transcriptome analysis resulted in 136 differentially expressed genes (DEGs) between spiderlings preying upon *N. lugens* fed on Bt- and non-Bt rice (Bt- and non-Bt spiderlings). Functional analysis indicated a potential impact of *Bt* toxin on the formation of new cuticles during molting. GO and KEGG enrichment analyses suggested that GO terms associated with chitin or cuticle, including “chitin binding”, “chitin metabolic process”, “chitin synthase activity”, “cuticle chitin biosynthetic process”, “cuticle hydrocarbon biosynthetic process”, and “structural constituent of cuticle”, and an array of amino acid metabolic pathways, including “alanine, asparatate and glutamate metabolism”, “glycine, serine and theronine metabolism”, “cysteine and methionine metabolism”, “tyrosine metabolism”, “phenylalanine metabolism and phenylalanine”, and “tyrosine and tryptophan biosynthesis” were significantly influenced in response to Cry1Ab.

**Conclusions:**

The Cry1Ab may have a negative impact on the formation of new cuticles during molting, which is contributed to the delayed development of spiderlings. To validate these transcriptomic responses, further examination at the translational level will be warranted.

**Electronic supplementary material:**

The online version of this article (doi:10.1186/s12896-016-0325-2) contains supplementary material, which is available to authorized users.

## Background

Genetically modified (GM) technology has reshaped the agricultural industry since its insertion in the late1990s [1]. From 1996 to 2012, the global acreage of GM crops has increased dramatically from 1.7 to 160.4 million hectares [2]. The ecological benefits from rapid development and adoption of GM crops include a significant reduction in both insecticide and herbicide usage and greenhouse gas emissions [3]. A meta-analysis in 2014 showed a 37% reduction in synthetic pesticide use, 22% increase in crop yield, and 68% increase in farmer profits [4].

Besides yield and profit gains and environmental benefits, non-monetary incentives include time savings, ease of use, and more flexibility in planning [5]. With limited arable land in China, GM technology provides a potential solution to improve agricultural productivity and sustainability. Currently, transgenic *Bacillum thuringiensis* (*Bt*) cotton, resistant to Lepidoptera pests, is the most successful commercial GM crop in China [6]. In 2012, acreage of Bt cotton has reached 3.59 million hectares, representing 80% of total cotton area in China. Bt cotton increased yield by 10%, reduced insecticide use by 60% and generated additional US $220 profit per hectare on average [7]. Even with the success of Bt cotton, consumers still have doubts about GM crops, partially due the lack of knowledge regarding the ecological risks [5, 8, 9]. Bt rice is facing the same challenges for the public acceptance.

The community structure of a rice field is primarily composed of soil organisms, rice, insect herbivores, predators, and parasitoids. While insect herbivores are exposed to *Bt* toxins by direct feeding, other community members can access *Bt* toxins through trophic interactions. Previous risk assessment studies showed no harmful effect of Bt rice on diversity, dominant species and abundance of non-target arthropods among the arthropod community in the field [10, 11]. Laboratory studies, on one hand, did not detect adverse impacts of Bt rice on non-target arthropods. For example, the developmental time, fecundity and survival rate of herbivorous insects *Nilaparvata lugens* and *Sogatella furcitera* were unaffected when exposed to Cry1C, Cry2A, and Cry1AC proteins, respectively [12, 13]. No significant effects were found on life history traits for predators as well, including *Chrysoperia sinica*, *Propylea japonica*, *Cyrtorhinus lividipennis*, and *Ummeliata insecticeps* [14–17]. On the other hand, some reports show non-target organisms may be susceptible to *Bt* toxins. A significant longer developmental time of *Pirata subpiraticus* was recorded when it prayed on Bt rice fed *Cnaphalocrocis medinalis* [18]. Significantly lower catalase activity was found in *Fosomia candida* fed on Bt rice in comparison to those fed on non-Bt rice [19]. Due to the varing degradation of *Bt* toxin protein in soils with different physicochemical properties [20, 21], researchers did not find consistent differences in soil microorganism communities between Bt and non Bt rice fields [22]. For parasitoids, effects of Bt rice is also inconsistent, which depends on the host species, target or non-target insects [22]. As a whole, risk assessment of Bt rice has been focusing on the organismal level impacts, suborganismal impacts are largely unknown. The advent of genomics era, however, allows us to evaluate ecological risks of transgenic Bt rice on non-target organism at the transcription and translational level.

The wolf spider *Pardosa pseudoannulata* is one of the dominant predators in South China, playing a crucial role in maintaining the stability of the rice agroecosystem [23]. In this study, we carried out a comparative transcriptome analysis of the 5th instar spiders fed on *N. lugens* maintained on Bt- and non-Bt rice, respectively. Developmental time from the 2nd to 8th instars was recorded to reveal the potential impacts of Bt rice on *P. pseudoannulata* and to correlate the biological impacts with differentially expressed genes.

## Methods

### Plant materials and *Nilaparvata lugens* preparation

Transgenic Shanyou 63 rice expressing Cry1Ab protein (test group) and its non-transgenic parental wild type Shanyou 63 rice (control group) were obtained from the Life Science College, Hunan Normal University. Both rice varieties were grown under nylon nets (3 × 2 × 1 m^3^) without insecticide application during the entire experimental period.

*Nilaparvata lugens* were collected from farmland in the Hunan Academy of Agricultural Science and reared on non-transgenic parental wild type, Shanyou 63, allowing for natural colonization. The newly moulted 2nd instar *N. lugens* nymphs were then transplanted to transgenic and control rice lines. After 15-day feeding, *N. lugens* was collected and used as spider diets [24]

### Spider sample collection

Female spiders with egg sacs were collected from the experimental farmland in the Hunan Academy of Agricultural Science. *Pardosa pseudoannulata* larvae were collected immediately after hatch and placed individually in a glass tube with a moist cotton ball separately (12 × 100 mm). Spiders in the test and control group were fed daily with *N. lugens* consumed Bt and non-Bt rice, respectively. All tubes were marked and maintained in an artificial climate chamber (30 °C, 70% RH and L:D 10:14 photoperiod). Developmental time of each spiderling at each instar was recorded until sexual maturity was reached. In this analysis, 120 spiders were raised for the developmental time recording (three biological replicates of 20 spiders each for 2 groups). Observation was made twice a day at 9 am and 9 pm, respectively.

### Quantification of the *Bt* toxin, Cry1Ab, in spiderling

An enzyme-linked immunosorbent assay (ELISA) was conducted for Cry1Ab protein detection using a Qualipate kit for Cry1Ab/Cry1Ac (EnviroLogix, US). For each treatment, five 5^th^ instar spiderlings were weighed as a group (test group, 0.0384 g, control group, 0.0316 g), homogenized in 1 ml PBS buffer and centrifuged for 20 min at 2,000 *g*. The supernatant was used to determine Cry1Ab concentration. Spectrophotometric measurements for three technical replications were obtained using a microplate reader (BioTek, ELX 800) at 450 nm. Purified Cry1Ab toxin (EnviroLogix, US) at concentrations of 0, 2, 4, 8, 16, and 24 ng/L was used to generate a standard curve. Three biological replicates were performed in ELISA assays.

### RNA isolation and Illumina sequencing

A total of ten 5^th^ instar spiderlings from the test and control groups were collected, respectively, on the ninth day after moulting and submitted to Oebiotech Enterprise (Shanghai) for RNA extraction and sequencing. Total RNA was extracted from each sample using TRIzol (Invitrogen Corp, USA) according to the manufacturer’s instructions. RNA quality was assessed using a NanoDrop ND-1000 spectrophotometer (Nanodrop Technologies Inc, Rockland, DE, USA) using a standard of1.8 ≤ OD260/OD280 ≤ 2.1 and was further confirmed by agarose gel electrophoresis.

RNA sequencing libraries were constructed and sequenced on flow cells using an Illumina Hiseq 2000 platform. Clean reads were assembled using the *de novo* transcriptome assembler Trinity after removing adaptor sequences, low quantity reads (reads with ambiguous bases N), and duplicate sequences [25]. The libraries were established and unigenes of length greater than 200 bp were subjected to subsequent sequence annotation analysis. All raw reads were deposited in the NCBI Sequence Read Archive (Accession number: SRR2024874, SRR2024877).

### Sequence annotation

All unigenes were compared to those available in the NCBI non-redundant protein (Nr) database and Swiss-prot database using Blastx with an E-value cutoff of 10-5. The Blast2GO program and WEGO software were used to obtain GO annotation for all unigenes [26, 27]. KEGG (Kyoto encyclopedia of genes and genomes database) metabolic pathway annotation and COG (clusters of orthologous group) classification of unigenes were determined by Blastx searching against KEGG and COG databases [28, 29]. The best aligning results were used to determine potential function of the unigenes.

### Identification of differentially expressed genes (DEGs)

The FPKM (number of reads per kb of exon region per million mapped reads) method was used for quantifying gene expression levels and was able to eliminate the influence of different gene lengths and sequencing levels in the calculation of gene expression [30]. The DEGseq software package (http://www.bioconductor.org/packages/2.6/bioc/html/DEGseq.html) was used to screen differentially expressed genes (DEGs) based on a statistical analysis of negative binomial distribution and to quantify the gene expression levels with baseMean values [31]. A threshold for false discovery rate of <0.01 and absolute value of log2 (fold change) ratio > 2 were used to determine significant differences in gene expression.

### Functional annotation of DEGs

All DEGs were searched against five public databases, Swiss prot, Nr, COG, GO, and KEGG. The hypergeometric test was used to find significantly enriched GO terms in DEGs based on GO annotation. The calculated *p* value then underwent Bonferroni Correction, using corrected *p* value ≤ 0.001 as a threshold. GO terms fulfilling this condition are defined as significantly enriched GO terms in DEGs. Similarly, pathway enrichment analysis was conducted to identify significantly enriched metabolic pathways or signal transduction pathways in DEGs, using *p* value ≤ 0.01 as a threshold.

### Quantitative real-time PCR analysis

Transcriptome results were verified using quantitative real-time PCR (qPCR). Total RNA was isolated from each sample with TRIzol (Invitrogen, USA) and subjected to DNase I treatment (Promega, USA) according to the manufacturers’ protocols. cDNA was synthesized with a RevertAid™ H Minus First Strand cDNA Synthesis Kit (Fermentas Lithuania) and qPCR was performed using the ABI 7900 HT system (ABI, USA) with a reaction volume of 25 μl containing 1 μl of 1:10 diluted cDNA in ddH2O, 12.5 μl of 2 × SYBR Green Master Mix (ABI, USA) and 100 nM of each of the primers. The qPCR conditions were 94 °C for 3 min, followed by 40 cycles of 94 °C for 30 s for denaturation, 55 °C for 30 s for annealing and 72 °C for 30 s for extension. The experiment was repeated three times, and expression levels of each gene were normalized to 18S ribosomal RNA (18S rRNA, GenBank accession number: X13457, primers: 5’-AGATGCCCTTAGATGTCCGG-3’, 5’-AAGGGCAGGGACGTAATCAA-3’). All primers were designed using the primer 3.0 program (http://bioinfo.ut.ee/primer3-0.4.0/) [32].

### Data analysis

Data on developmental time of spiderlings and qPCR were analysed using a *t*-test with SPSS 17.0 software. Significant differences at *p* < 0.05 were designated with *, and data were presented as the mean ± SE.

## Results

### Bt rice affects the developmental time of *P. pseudoannulata*

**Fig. 1:**
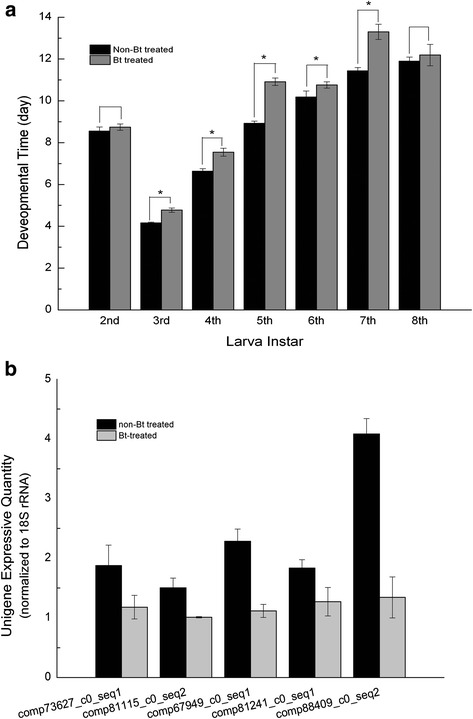
Developmental time of *P. pseudoannulata* spiderling. **a** The *left column* showed developmental time of spiderlings in the control group, and the *right column* showed that from the test group. *t*-test was used to determine the significance (*p* < 0.05). **b** qPCR analysis. Technical replicates were performed for each of three biological replicates. Data were presented as mean ± SE

The Cry1Ab protein content of 5th instar *P. pseudoannulata* spiderlings fed on *N. lugens* maintained on Cry1Ab rice was 1.451 ng/g, while no detectable level of Cry1Ab was observed in the spiderlings fed on *N. lugens* maintained on wild type rice. For total developmental time, the Bt spiderlings spent more time to reach the mature stage when compared to the controls (control group: 67.2 ± 1.58 days, test group: 73.2 ± 1.022 days, *p* < 0.05). The intermoult period of each instar in Bt spiderlings was also longer than that of controls at each instar, except for the 2nd and 8th (*p* < 0.05) (Fig. 1a).

### Illumina sequencing and de novo assembly

Sequences of mRNAs pooled from the whole body of spiderlings were analyzed using an Illumina 2000 platform and resulted in 48,243,314 and 42,798,756 raw reads for non-Bt and Bt spiderlings, respectively. Trinity software was used for de novo assembly according to standard parameters. The assembly yielded 217,017 total transcripts (≥200 bp) with an average length of 612 bp, and the unigene dataset included 169,703 sequences with an average length of 537 bp. All unigenes were used for the annotation.

### Annotation of all assembled unigenes

A total of 169,703 unigene sequences were subjected to blast searching against five public available databases, including Nr, Swiss-prot, COG, GO and KEGG, with a cutoff E < 10^−5^. Of these, 39,727 (23.4%) could be matched to Nr, 31,039 (18.3%) to Swiss-prot, 7,111 (4.2%) to KEGG, 28,646 (16.9%) to COG and 33,652 (19.8%) unigenes to GO database.

**Fig. 2:**
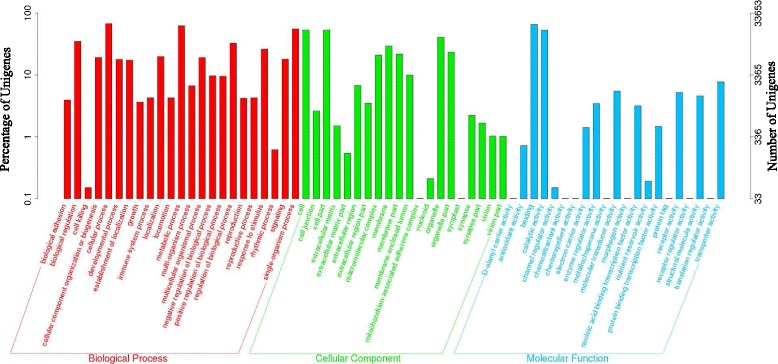
GO classification for all unigenes of *P. pseudoannulata* spiderling. GO categories shown on the *X*-axis were grouped into three main ontologies: biological process, cellular component and molecular function. The right *Y*-axis indicated the number of genes in each category while the left *Y*-axis represented the percentage of genes in that category. The Blast2GO program was used to obtain GO annotation of all unigenes (level 2)

**Fig. 3:**
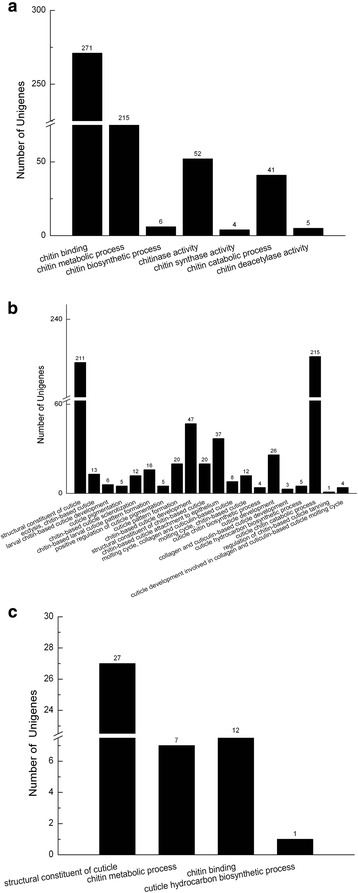
GO categories associated with chitin synthesis or cuticle formation. **a** GO categories related to chitin synthesis. **b** GO categories related to cuticle formation. **c** DEGs associated with chitin synthesis and cuticle formation between Bt- and non-Bt spiderdlings. A unigene may be placed in more than one group increasing the total number of genes

The ontology covers three domains: cellular component, molecular function and biological process [33]. Based on similarity search, 33,652 sequences among all uingenes were categorized into 10,759 GO terms consisting of three domains: cellular component category (1,073), molecular function (2,663) and biological process (7,023 unigenes) and a few fell into more than one gene function group (Fig. 2). Most sequences were annotated as “cellular process”, “single-organism process”, “metabolic process” and “binding”. The results from GO classification presented a portion of unigenes that had the potential to impact molting, a necessary process for *P. pseudoannulata* to grow. Specifically, many unigenes corresponded to seen chitin-related GO terms (Fig. 3a) and 20 cuticle-related GO terms (Fig. 3b), including 271 in the category of “chitin binding”, 215 in “chitin metabolic process” , 215 in “cuticle chitin catabolic process”, and 211 in “structural constituent of cuticle”.

**Fig. 4:**
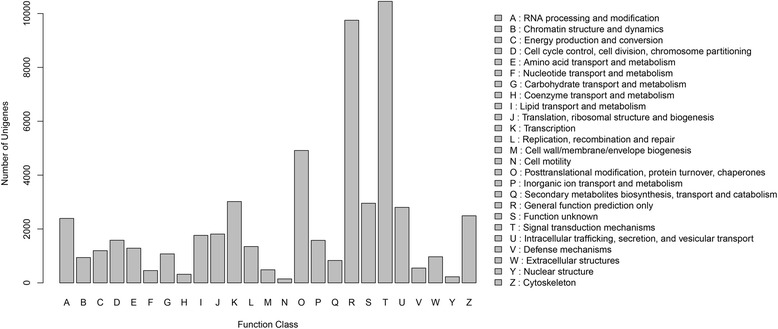
Histogram of COG classification. A total of 28,646 unigenes were assigned to 25 categories in the COG classification. The right legend shows a description of the 25 function categories. Some of them can be placed in more than one COG

COG is a database where orthologous genes are classified. Twenty eight thousand six hundred forty-six unigenes among all unigenes were classified and divided into 25 COG categories, among which a cluster for “Signal transduction mechanisms” represented the largest group (10,452, 36.5% of all matched unigenes) followed by “General function prediction only” (9,750, 34.0%) and “Posttranslational modification, protein turnover, chaperones” (4,915, 17.2%). The categories of “Cell motility” (0.5%), “Nuclear structure” (0.8%) and “Coenzyme transport and metabolism” (1.1%) had the fewest matching genes (Fig. 4). A group of 66 unigenes were assigned to the category of “chitinase”, which is responsible for dissolving the endocuticle during molting.

### Identification of DEGs and functional analysis

**Fig. 5:**
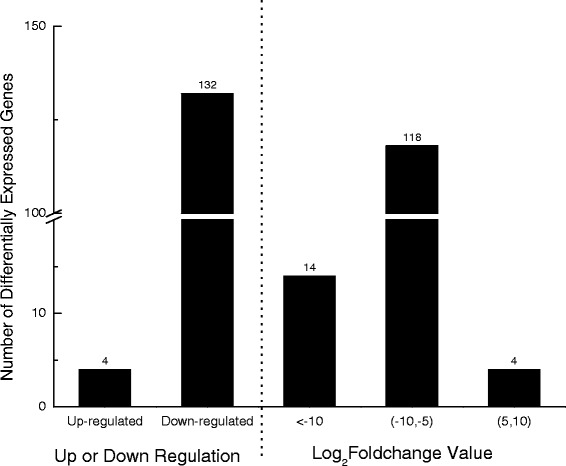
Distribution of differentially expressed unigenes between Bt- and non-Bt spiderlings. The threshold was based on FDR < 0.001 and absolute value of log2 ratio >2.

In total, 136 DEGs, including 132 down- and four up-regulated, were detected in the Bt spiderlings, compared to the controls (Additional file 1: Table S1). Among all DEGs, 122 had a fold change ranging from five to ten and the remaining 14 had a fold change of more than ten (Fig. 5). For all DEGs, 76 (55.9%) were assigned to Nr, 55 (40.4%) to Swiss prot, 18 (13.2%) to COG, 9 (6.6%) to KEGG and 60 (44.1%) to GO database. Interestingly, several DEGs were found to regulate biological processes associated with cuticle or chitin, including 27 down-regulated DEGs that were categorized to the GO term “structural constituent of cuticle”, 12 to “chitin binding”, 7 to “chitin metabolic process” and 1 to “cuticle hydrocarbon biosynthetic process”, implying a potential effect of Cry1Ab on spiderling molting (Fig. 3c).

**Fig. 6:**
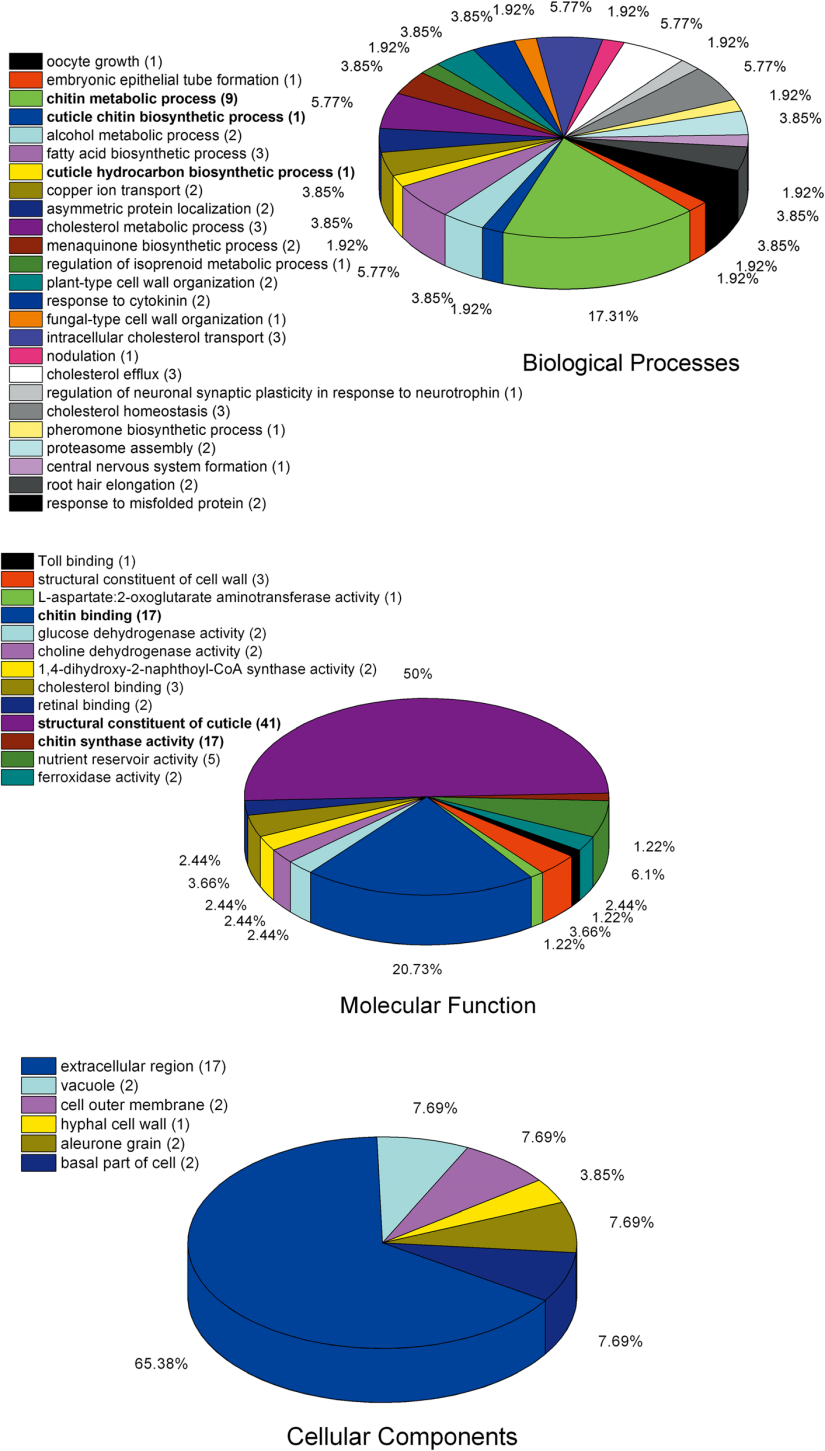
Categories of significantly enriched GO terms within DEGs between Bt and non-Bt spiderdlings. Numbers in brackets indicated the sum of annotated DEGs

The clusters of DEGs were further characterized by GO and KEGG enrichment analyses, respectively [34]. DEGs were significantly enriched to 44 GO terms (FDR < 0.001), including six belong to “Cellular component” (26 unigenes), 13 to “Molecular function” (82 unigenes) and 25 to “Biological process” (52 unigenes) (Fig. 6). Six of the 44 enriched GO terms were related to chitin and cuticle, including three terms which belong to molecular function categories, “structural constituent of cuticle”, “chitin binding” and “chitin synthase activity”, and three to biological process, “chitin metabolic process”, “cuticle chitin biosynthetic process” and “cuticle hydrocarbon biosynthetic process” (Bold in Fig. 6). Metabolic pathway enrichment analysis demonstrated that 136 DEGs were involved in 20 pathways (Table 1). Among them, 16 functional pathways were significantly enriched (FDR < 0.01), seven of which have been associated with amino acid metabolism, including Alanine, asparatate and glutamate metabolism (ko00250); Glycine, serine and theronine metabolism (ko00260); Cysteine and methionine metabolism (ko00270); Tyrosine metabolism (ko00350), Phenylalanine metabolism (ko00360); Phenylalanine, tyrosine and tryptophan biosynthesis (ko00400) and Biosynthesis of unsaturated fatty acids (ko01040). Apparently, various amino acid metabolic pathways in *P. pseudoannulata* spiderlings were affected in response to Cry1Ab

**Table 1:** Enriched pathways of DEGs between Bt- and non-Bt spiderdlings

ID	Term	FDR
ko00062	Fatty acid elongation	0.0005845
ko00250	Alanine, aspartate and glutamate metabolism	0.0075729
ko00260	Glycine, serine and threonine metabolism	0.0086477
ko00270	Cysteine and methionine metabolism	0.0066051
ko00330	Arginine and proline metabolism	0.0103575
ko00350	Tyrosine metabolism	0.0031269
ko00360	Phenylalanine metabolism	0.0031269
ko00400	Phenylalanine, tyrosine and tryptophan biosynthesis	0.0007844
ko00650	Butanoate metabolism	0.0037787
ko00710	Carbon fixation in photosynthetic organisms	0.0041007
ko00950	Isoquinoline alkaloid biosynthesis	0.0008605
ko00960	Tropane, piperidine and pyridine alkaloid biosynthesis	0.0007844
ko01040	Biosynthesis of unsaturated fatty acids	0.0031269
ko01200	Carbon metabolism	0.0421923
ko01210	2-Oxocarboxylic acid metabolism	0.0031269
ko01212	Fatty acid metabolism	0.0121062
ko01230	Biosynthesis of amino acids	0.0246187
ko03320	PPAR signaling pathway	0.0070911
ko04142	Lysosome	0.0007775
ko04978	Mineral absorption	0.0005845

### Real-time PCR assays

To validate RNA-seq results, five DEGs were randomly selected for qPCR analysis. These genes were homologous to structural constituents of cuticle, chitin binding and chitin metabolic process (Additional file 2: Table S2). The expression profile of all five DEGs was consistent with RNA-seq data (Fig. 1-b).

## Discussion

*Bt* toxins can be transferred via the food web and accumulate in organisms to different degrees [35]. The level of *Bt* toxin protein in predators mainly depends on expression patterns of *Bt*-protein in plants, and the feeding behavior of the herbivore [36]. Our tritrophic bioassay indicated the accumulative Cry1Ab content in 5^th^ instar spdierling was 1.451 ng/g when *P. pseudoannulata* was preyed on *N. lugens* maintained on Cry1Ab rice. Although this protein level is slightly lower than those in *Ummeliata insecticeps* (2.04 ng/g) [37], it is still informative. Developmental time of Bt spiderlings was significantly prolonged, which is consistent with *Pirata subpiraticus* [35]. However, spiderlings were able to recover from the effect of Bt rice at a later instar. Similar to other arthropods, *P. pseudoannulata* must molt periodically to grow. The formation of new cuticle is a vital step during molting of arthropods [38]. We speculated that the delayed development of spiderlings may be due to the disruption of chitin synthesis (formation of the new cuticle) during molting.

Comparative transcriptome analysis identified 136 DEGs between Bt- and non-Bt spiderlings (FDR < 0.001, Log2foldchange > 2). Furthermore, GO annotation and enrichment analysis both suggested potential impacts of Bt rice on the chitin synthesis and cuticle formation (Fig. 3-c, Fig. 6). As with other arthropods, the exoskeleton of spider is made of cuticle, of which one of the primary component is chitin [39]. The molting process in spiders involves activation of hypodermal cells, secretion of exuvial fluid and apolysis, activation of enzymes in the exuvial fluid, and secretion of the new cuticle [40]. Functional analysis of DEGs suggested a disruption of new cuticle formation during molting. In addition, GO and KEGG enrichment analyses indicated that GO terms associated with chitin or cuticle, including “chitin binding”, “chitin metabolic process”, “chitin synthase activity”, “cuticle chitin biosynthetic process”, “cuticle hydrocarbon biosynthetic process”, and “structural constituent of cuticle”, and an array of amino acid metabolic pathways, including “alanine, asparatate and glutamate metabolism”, “glycine, serine and theronine metabolism”, “cysteine and methionine metabolism”, “tyrosine metabolism”, “phenylalanine metabolism and phenylalanine”, and “tyrosine and tryptophan biosynthesis” were significantly affected in response to Cry1Ab.

The advent of Genomic Era offers new transcriptome resources for the study of wolf spiders. Meng et al. sequenced cephalothoraxes of *P. pseudoannulata* adults and identified genes involved in insecticide metabolism and detoxification, including P450s, GSTs, AChEs, AChRs, GABA receptors, and GluCI [41]. Xiao et al. carried out RNA-Seq analysis in *P. pseudoannulata* and revealed an array of genes responding to temperature stress [42]. In this study, we focused on the genes corresponding to ingested *Bt* toxins. As a non-model animal without a reference genome, omics resources, such as transcriptomes, lay the foundation for future functional genomic research.

## Conclusions

The Cry1Ab may have a negative impact on the formation of new cuticles during molting, which is contributed to the delayed development of spiderlings. To validate these transcriptomic responses, further examination at the translational level will be warranted.

## Additional files

Additional file 1: Table S1. DEGs between Bt and non-Bt spiderlings. In all 136 DEGs, including 132 down- and 4 up-regulated, were detected in the Bt spiderlings, compared to the controls. (DOCX 16 kb)
Additional file 2: Table S2. Selected DEGs for qPCR and their primers. These genes were homologous to structural constituents of cuticle, chitin binding and chitin metabolic process. (XLS 55 kb)

## Abbreviations

BPHs: Brown planthoppers
Bt: *Bacillus thuringiensis*
COG: Cluster of Orthologous Groups of proteins
DEG: Differential expression genes
FPKM: Number of reads per kb of exon region per million mapped reads
GM: Genetically modified
GO: Gene ontology
KEGG: Kyoto Encyclopedia of Genes and Genomes Database
PBS buffer: Phosphate buffer solution
qPCR: Real-time PCR
RNA-Seq: RNA sequencing
