# Supplementary material for: Transcriptomic response of wolf spider, Pardosa pseudoannulata, to transgenic rice expressing Bacillus thuringiensis Cry1Ab protein
Source: BMC Biotechnol. 2017 Jan 18;17:7. doi: 10.1186/s12896-016-0325-2 (PMC5241980; doi:10.1186/s12896-016-0325-2)
Supplement: Additional file 1: Table S1. — DEGs between Bt and non-Bt spiderlings. In all 136 DEGs, including 132 down- and 4 up-regulated, were detected in the Bt spiderlings, compared to the controls. (DOCX 16 kb) [file 12896_2016_325_MOESM1_ESM.docx]

Additional file 1: Table S1. Selected DEGs for qPCR and their primers

| Unigene | Size (bp) | Similarity | Primer Sequence |
| --- | --- | --- | --- |
| comp73627_c0_seq1 | 1000 | structural constituent of cuticle  chitin binding | F:ACCATAAGCAGCCACTGGTC  R:CAGTCGTCAGGACCAACGAA |
| comp81115_c0_seq2 | 1215 | structural constituent of cuticle | F:TCCTCTACAAGAGCCGGTCA  R:CCGTAGGTGGGTACTCCAGA |
| comp81241_c0_seq1 | 1396 | chitin metabolic process  chitin binding | F:GGTTTCCGCAAAGGAAGCTG  R:ACGAACCAATTTTGCCTGCC |
| comp88409_c0_seq2 | 1620 | structural constituent of cuticle | F:GGCGTTCCCAATCCTTACCA  R:TGTCGAACACGAATGGCTGA |
| comp67949_c0_seq1 | 1463 | structural constituent of cuticle | F:TCGGACAATCTGGACAAGCC  R:GACGTATGGGTAGACAGCGG |
